# Anthropogenic edge effects and aging errors by hunters can affect the sustainability of lion trophy hunting

**DOI:** 10.1038/s41598-022-25020-9

**Published:** 2023-01-12

**Authors:** Andrew J. Loveridge, Matthew Wijers, Roseline Mandisodza-Chikerema, David W. Macdonald, Guillaume Chapron

**Affiliations:** 1 Trans-Kalahari Predator Programme, WildCRU, Recanati-Kaplan Centre, Department of Zoology, University of Oxford, Oxford, UK; 2 Zimbabwe Parks and Wildlife Management Authority, Cnr. Borrowdale Road/Sandringham Drive, Alexandra Park, P.O. Box CY 140, Harare, Zimbabwe; 3 Institutionen för Ekologi, Grimsö Wildlife Research Station, Swedish University of Agricultural Sciences, Riddarhyttan, Sweden

**Keywords:** Ecology, Behavioural ecology, Conservation biology, Population dynamics

## Abstract

Many large predator populations are in decline globally with significant implications for ecosystem integrity and function. Understanding the drivers of their decline is required to adequately mitigate threats. Trophy hunting is often cited as a tool to conserve large mammal populations but may also have negative impacts if not well managed. Here we use a spatially implicit, individual based model to investigate the threats posed to African lion populations by poorly managed trophy hunting and additive anthropogenic mortality such as poaching and retaliatory killing. We confirm the results of previous studies that show that lion trophy hunting can be sustainable if only older male lions are hunted, but demonstrate that hunting becomes unsustainable when populations are exposed to additional anthropogenic mortality, as is the case for most free ranging populations. We show that edge effects can be a critical determinant of population viability and populations that encompass well protected source areas are more robust than those without. Finally, errors in aging of hunted lions by professional trophy hunters may undermine the sustainability of the age-based quota setting strategies that are now widely used to manage lion trophy hunting. The effect of aging errors was most detrimental to population persistence in the ≥ 6 and ≥ 7 year-old age thresholds that are frequently used to define suitably aged lions for hunting. Resource managers should limit offtakes to older demographics and additionally take a precautionary approach when setting hunting quotas for large carnivore populations that are affected by other sources of anthropogenic mortality, such as bush-meat poaching, retaliatory killing and problem animal control.

## Introduction

Globally, biodiversity is in decline, largely as a result of anthropogenic pressures on the biosphere^1,2^ and many species face extinction^3^. This is particularly true of large predators that are reliant on relatively intact ecosystems and prey assemblages to survive^4^, but also because predator species are often directly exploited or persecuted by people. As a result many large predator populations are in severe decline^5^. At the same time ecosystems that lose their apex predators have been shown to be less resilient and more prone to ecosystem collapse and trophic downgrading^6^.

African lions (*Panthera leo*) are a prime example of this trend. The species geographic range in Africa has contracted significantly compared to historical distributions, continental populations totaled only ~ 25 000 lions in 2015, with a 50% decline predicted over the next two decades^7^. Causes of decline are thought to be primarily due to loss of habitat to agriculture, declining prey populations, conflicts with livestock farmers and in some cases over-exploitation of populations by trophy hunting and illegal killing for body parts^8^.

Use of trophy hunting as a conservation strategy is widely practiced in Africa^9^, although hunting of charismatic species such as lions has become a contentious issue^10^. Critics argue that trophy hunting is morally reprehensible, imperils already declining species and is frequently poorly managed, provides little benefit to rural Africans^11,12^ and is, in some cases, beset by corrupt practices^13^. However, proponents of trophy hunting stress, that if well managed, the practice protects wildlife habitat from conversion to agriculture and generates revenue for conservation and local economies thereby providing a justification for continued conservation of wild species and habitats^14^.

Trophy hunting of lions has been shown to be sustainable under strict conditions^15^, although several intensive field studies have also shown that unsustainably managed trophy hunting of lion populations can precipitate population declines^16–18^. Indeed, sexually-selected infanticide, which occurs frequently following a pride takeover, amplifies mortality^18^. Therefore, there is a clear need for trophy hunting management to be underpinned by a strong scientific basis for determining sustainability of legal hunting offtakes.

Lion conservation management has been informed by several important theoretical studies which have modelled lion population dynamics in the context of population management. Early models demonstrated the importance of incorporating social behavior and territoriality into population simulations for lions and were used as the basis to simulate population control regimes (including culling and contraception) in southern African protected areas^19–21^. This modelling approach, hereafter referred to as the Starfield model, was further developed as an individual based, spatially explicit demographic model^15^, parameterized with detailed demographic data from the Serengeti National Park to demonstrate the effects on population size of super-additive mortality caused by unsustainable trophy hunting of territorial male lions. That study proposed that trophy hunting is sustainable if only male lions aged a minimum of 6 years old are hunted. This recommendation has been widely adopted by resource managers in range states where lion trophy hunting occurs^22^.

These concepts were further developed by Creel et al.^23^, using a Leslie matrix approach (hereafter referred to as the Creel model) and parameterized with population data from the Luangwa NP, Zambia. The Creel model confirmed that hunting only older males ensures population sustainability but recommended that the minimum age of trophy hunted males should be at least 7 years and that periods of population recovery be incorporated into trophy hunting management strategies.

The various iterations of the Starfield model were parameterized with data from stable, highly protected populations (Serengeti and Kruger National Parks) where age dependent mortalities from anthropogenic threats were low or non-existent^24^ and therefore models did not incorporate additional anthropogenic mortality, such as deaths due to retaliatory killing by livestock owners, problem animal control and wire snare poaching. However, these anthropogenic sources of mortality are common and increasingly important threats to the persistence of many lion populations^25,26^. Demographic data from Luangwa National Park used in the Creel model, share characteristics with many less well protected populations that are exposed to higher levels of anthropogenic threat. This model implicitly included other sources of anthropogenic mortality in population survival rates used but did not explicitly examine or manipulate these effects.

Neither the Starfield nor Creel models incorporated the effects of spatially heterogeneous mortality, assuming instead that all individuals in the population of appropriate age were equally exposed to mortality from trophy hunting. Whilst this is a realistic scenario in hunting blocks where hunters can access the entire population, trophy hunting frequently occurs on or close to the boundaries of fully protected areas such as national parks, and exerts a significant edge effect on these areas^18,27,28^. Loveridge et al.^29^ described such an effect in Hwange National Park, where trophy hunting of male lions in hunting blocks adjacent to the national park boundary created territorial vacancies in peripheral areas which were sequentially filled by new males from the national park core. This exposed a high proportion of lions in the protected population to trophy hunting mortality. This was termed the vacuum effect and it is likely this source-sink dynamic is common where shared populations are fully protected in national parks but exploited in surrounding hunting concessions.

Management of trophy hunting quotas using minimum age-based thresholds is predicated on the ability of professional hunters to accurately age animals before hunting them. Using photographs of known age lions from field studies, Miller et al.^30^ assessed the ability of experienced professional hunters to accurately age males across different age categories. Hunters were able to accurately discern younger lions (< 3 years old) from older animals and were more successful at aging older animals (> 7 years). However, they were less able to reliably age lions between 4 and 7 years old, with a tendency to over-estimate ages within these groups. This is potentially problematic in that many age-based hunting strategies recommend limiting offtakes to animals ≥ 6 years^14^ or ≥ 7 years^23^, with hunters expected to assess suitability of trophies falling into age categories where age estimation, by even experienced practitioners, is least accurate.

In order to take these additional considerations into account, we developed an individual based, spatially implicit, population demographic model, parameterized with data from a 20-year study in and around Hwange National Park, Zimbabwe. To realistically reflect the conditions prevalent in many contemporaneous protected area populations and the practicalities of sustainably managing large predator populations, we explicitly examined the impacts of multiple forms of anthropogenic mortality (trophy hunting, conflict with livestock owners and poaching) in order to assess how these impacts might affect the sustainability of trophy hunting. Furthermore, we modelled the effects of spatially heterogeneous anthropogenic mortality to simulate anthropogenic edge effects, sensu Woodroffe and Ginsberg^31^, and source-sink dynamics experienced by populations of many large carnivores in protected areas^32^. Finally, we tested the effects of misclassification of lion ages by professional hunters in the field on the predictions and sustainability of minimum-age hunting strategies.

## Results

### Simulation of edge effects

**Figure 1:**
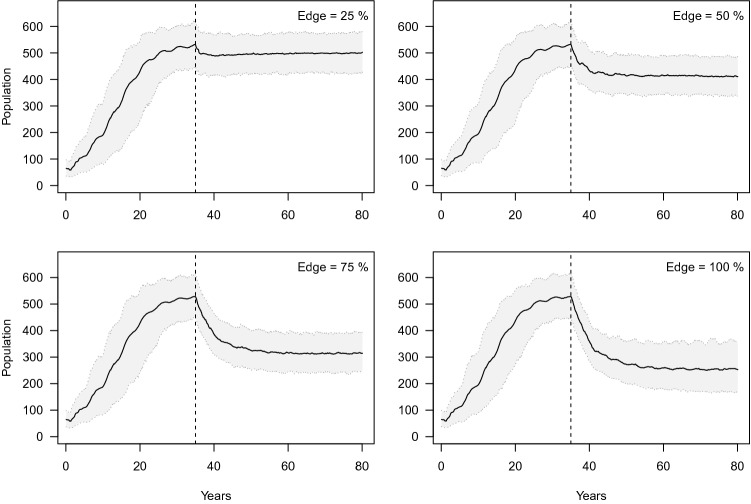
Effect of hunting (annual hunting quota = 18 males ≥ 6 years) and conflict (0.4% monthly mortality rate) on lion population persistence with varying edge effects (% of prides exposed to conflict and hunting; see Figure S4). The continuous black line represents the mean and the light grey area the 95% confidence interval. The vertical dotted line marks the point at which test scenarios were implemented after the population had reached its asymptotic state.

Increasing proportions of prides affected by edge effects and thus smaller protected source populations resulted in concomitantly larger declines in population size. When 25% of prides were affected by edge effects there were small declines (6%) in population size after 45 years. A 23% decline was recorded when half of prides were affected by edge effects and 40% and 52% declines resulted, respectively, when 75% and 100% of prides were affected (Fig. 1). These effects were exacerbated when younger minimum male age threshold hunting strategies were used (Figure S3). In addition, larger edge effects resulted in increasingly female biased adult sex ratios as well as declines in total number of individuals, prides, adult females, cubs and total number of males that could be trophy hunted in ‘edge’ areas (Figure S4, S5).

### Simulation of additive effects of trophy hunting and other anthropogenic mortality

**Figure 2:**
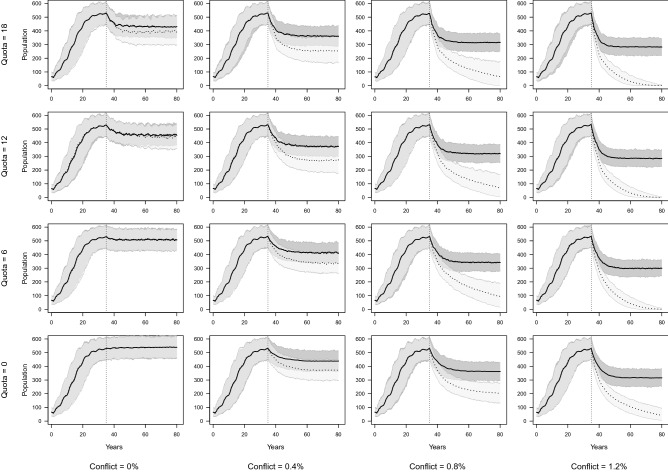
Effect of varying hunting quotas and conflict mortality rates on lion population persistence. Solid line: 25 of 40 (63%) prides vulnerable to edge effects (hunting and conflict), approximating the impacts experienced by the HNP population. Dotted line: All 40 prides in the simulation impacted by hunting and conflict mortality. Only male lions, ≥ 6 years old were eligible to be hunted. The black lines represent the mean and the light grey areas the 95% confidence interval of simulations. The vertical dotted line marks the point at which test scenarios were implemented after the population had reached its asymptotic state.

Simulated populations with a well-protected core source population and fewer prides exposed to edge effects (37% of prides) were markedly more resilient than populations with no protected source under the same hunting and conflict regimes (Fig. 2, Table S7a). In all scenarios with a protected core, populations remained stable over the final 21 years, or three lion generations^7^ of the simulation, though declined from initial population levels in scenarios that included hunting or conflict mortality. Over 45 years, populations in scenarios that included hunting, but no conflict declined by 20% or less and those that included low or moderate levels of hunting and low levels of conflict by less than 30%. Moderate and high conflict as well as high hunting and low conflict mortality resulted in population declines of 30% or more.

In contrast, for populations with no protected core source population, all scenarios that included trophy hunting and low levels of conflict mortality declined by 38–52% over 45 years depending on the size of hunting quota. Scenarios with hunting and moderate levels of conflict, declined precipitously by more than 85% while those with high levels of conflict were extirpated over the same period (Fig. 2, Table S7b). In simulations with moderate or high conflict and no hunting populations declined by > 60% and > 90% respectively. Only low hunting offtakes in the absence of conflict mortality resulted in declines < 10%.

The percentage of failed hunts increased with both hunting quotas and levels of conflict mortality, reaching close to 100% failure with high levels of conflict and high hunting quotas in the absence of a protected core area (Figure S5a and b).

### Minimum age-based thresholds for trophy hunting offtakes

Simulations of minimum age-based hunting thresholds ranging from ≥ 3 to ≥ 8 years, confirm the findings of the Starfield and Creel models showing that trophy hunting young and prime age male lions is severely deleterious to populations, whilst restricting offtakes to only older males was more sustainable. In all scenarios, in the absence of a protected core (Figure S6a), populations declined from initial levels with the introduction of hunting. Scenarios with no additional conflict mortality resulted in relatively small impact to the population (13–14% declines over 45 years) when age thresholds were ≥ 7 years and above. The negative effects of hunting offtakes were amplified by increasing levels of conflict mortality with the steepest population declines occurring when age thresholds were low and conflict mortality was moderate or high. Scenarios where age thresholds were ≥ 6 years, and/or where conflict occurred, populations declined by more than 30% over 45 years. Populations declined to extinction in scenarios with moderate conflict and ≥ 5 year age thresholds and below, and in all scenarios with high levels of conflict mortality. Only ≥ 7 and ≥ 8-year minimum age thresholds resulted in stable populations in scenarios with low levels of conflict mortality, with ≥ 8-year-old thresholds clearly the least impactful on the population. In all scenarios with moderate and high conflict mortality, populations declined regardless of the minimum age threshold. The presence of a protected core population ameliorated the impacts of both higher levels of conflict mortality and lower minimum age thresholds (Figure S6b).

### Simulation of hunter error in field age assessments

**Figure 3:**
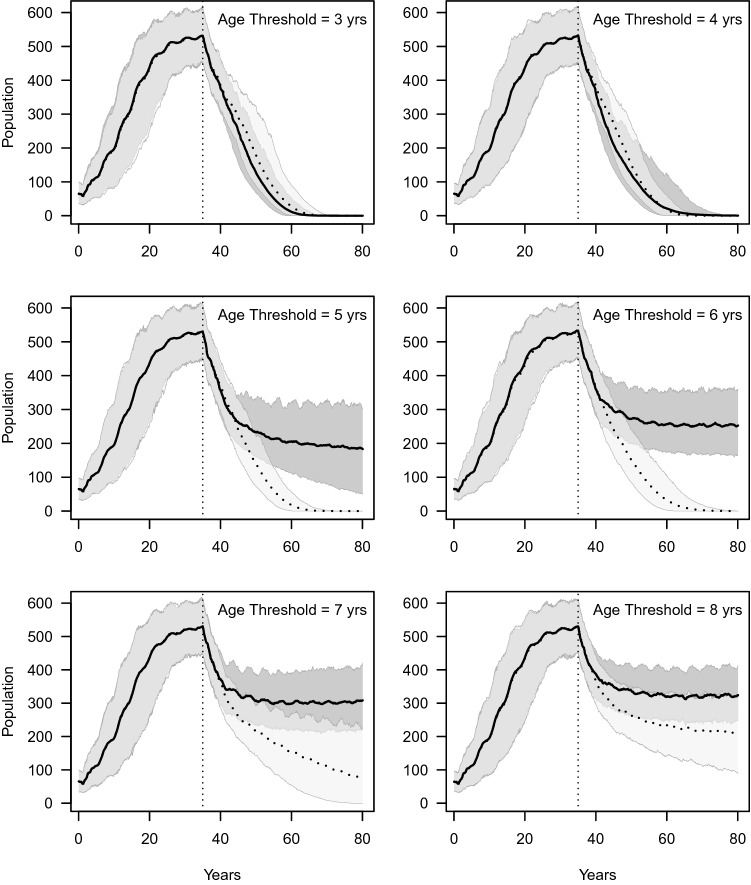
Effect of unreliable age assessment of hunted lions by professional hunters on lion population persistence for minimum age thresholds of ≥  3 to ≥ 8 years (based on ageing data reported in^30^). Simulations used an annual hunting quota of 18 males (~ 1.2 lions/1000 km^2^) and a monthly conflict mortality rate of 0.4% with no edge effect. Curves with solid lines denote scenarios where ‘real’ ages of hunted lions were derived from the model and dotted lines incorporate age specific errors in age estimation by hunters. The black lines represent the mean and the light grey areas the 95% confidence interval of simulations. The vertical dotted line marks the point at which test scenarios were implemented after the population had reached its asymptotic state. After 45 years, populations in estimated age scenarios were the same or smaller than actual age scenarios (% difference between actual and estimated: ≥ 3 = 0, ≥ 4 = 0, ≥ 5 = − 100, ≥ 6 = − 99.9, ≥ 7 = − 80.0, ≥ 8 = − 33.6). See Figure S6 and S7 for expanded scenarios testing the combined effect of unreliable age assessment, edge effects and conflict mortality.

Where no protected population core was present, impact of errors in aging as evidenced by the difference between ‘actual’ and ‘estimated’ age simulations in each minimum age threshold was negligible for age thresholds ≥ 3, ≥ 4 years, though neither age threshold was sustainable (Fig. 3). For all other minimum age threshold hunting quota strategies ‘estimated’ age scenarios resulted in smaller populations than those predicted by ‘actual’ age scenarios due to overestimation of ages by professional hunters. This discrepancy was most severe in the ≥ 6 year and ≥ 7 year age groups where ‘estimated’ age scenarios resulted in population extirpation. With the exception of the ≥ 8 year old minimum age threshold scenario all ‘estimated age’ scenarios resulted in steep population decline over the simulated period of 45 years. For the ≥ 8 year old threshold scenario, populations declined less steeply but resulted in a ~ 30% smaller population for ‘estimated’ compared to ‘actual’ scenarios The effects of aging errors were exacerbated by higher levels of conflict mortality but eased by the presence of a protected population core (Figure S6, S7).

## Discussion

Many large predator populations are in decline globally because of an expanding human footprint on natural ecosystems. It is critical to understand the influence of anthropogenic threats on population dynamics. African lions are well studied, providing a tractable model species to examine the spatial and demographic impacts of anthropogenic threats on large predator populations in protected areas. In addition, they are a species requiring urgent conservation attention as populations are declining both numerically and geographically. Lions are also commercially exploited through trophy hunting, the regulation of which is of concern to both range states and hunting trophy importing nations. Information on sustainability of hunting strategies is required to ensure compliance with international conventions and trade agreements^33^ and to demonstrate non-detriment and conservation enhancement to hunted populations^34^.

Our simulations show that trophy hunting mortalities when occurring simultaneously with other sources of anthropogenic mortality, such as bush-meat snaring and retaliatory killing, on the boundaries of protected areas can have profoundly detrimental impacts and potentially compromise population viability. This aligns with previous research showing that the impact of edge effects on lion populations results in reduced pride size, pride survival^35^, individual adult survival, cub survival^18^ and sub-adult disperser survival^36^ compared to populations or sub-populations free of these threats. Similarly, Packer et al.^37^ show that fenced populations (that experience few edge effects) are highly likely to persist whereas unfenced populations that are exposed to edge effects were likely to decline to extinction in more than half of cases examined. Large predator populations are increasingly isolated as human population growth and agricultural expansion increase^1^ and even when protected by national parks are still exposed to mortality on park boundaries^27,28,38^. For a simulated lion population, with 25 of 40 prides exposed to edge effects (approximating the situation in unfenced HNP), the combination of even low levels of trophy hunting and low to moderate levels of additional anthropogenic mortality on the PA boundary is enough to precipitate undesirable population declines of between 23 and 36 percent. For National Park populations, where one of the primary purposes is to protect biodiversity and intact ecological processes, the consequences of such declines in apex predator populations may be detrimental disruption of trophic systems and ecosystem function^5,6^. Furthermore, because lions are a major attraction in the African photographic tourism industry, declines in lion populations could negatively impact marketability of the tourism product^39^.

Nevertheless, populations in protected areas experiencing only moderate edge effects are significantly more resilient to perturbations than those in which anthropogenic perturbations permeate the entire protected area. This supports the suggestion that source-sink meta-population dynamics^40^ are critical in determining the persistence of predator populations across contemporary conservation landscapes. Movements of animals from source into sink habitats have been found to ameliorate the effects of site specific anthropogenic mortality at meta-population scales, and to be critical for persistence of local predator populations outside source areas (e.g. brown bears-*Ursus arctos*^41^; cougars-*Puma concolor*^42^; leopards-*P. pardus*^38^). However, source sink dynamics may also obscure the severity of local scale mortality (due to movement of animals from source to sink) and potentially confound management outcomes if inadequately accounted for when planning regional scale consumptive use or population management^43^.

For a fully protected lion population, impacted on its edges but providing a core refuge for part of the population, the core source or refuge population buffers the sink effect on the population’s boundary. Conversely, where perturbations affect the entire population (such as in a small isolated protected area, an area designated for hunting, or one heavily affected throughout by poaching) the absence of a core source can result in profound and sustained population declines. In our model, under these circumstances, simulated populations declined to extinction in scenarios with hunting and even moderate levels of anthropogenic mortality. Source-sink meta-population management for large wide ranging predators can be facilitated by protecting extensive habitat (such as large national parks or national parks buffered by well managed hunting areas) or enhancing connectivity corridors or habitats between neighboring protected populations to allow movement between them, as has been suggested for the lion population in the KAZA region^44^.

Adult survival and fecundity are critical in determining population trends in long-lived vertebrates^45^, and age is a common metric used to guide sustainable offtakes from wildlife populations^46^. However, the accuracy of field aging techniques has been shown to vary widely for several mammalian herbivore^47,48^ and carnivore species^49–51^. Misclassification of age classes used in resource management decision making can undermine the effectiveness of conservation programmes.

Management of lion trophy hunting is frequently on the basis of minimum age thresholds, typically limiting offtakes to males ≥ 6 years old^15^, with quotas adapted according to the consistency of offtakes above or below this threshold^52,53^. Our findings with regard to sustainability of trophy hunting support the findings of previous studies^15,23^ showing that hunting quotas limited to older age classes are more sustainable. However, hunting management strategies based on minimum age thresholds rely heavily on both reliability of field assessment of lion ages by professional hunters and subsequent age verification, through examination of photographs and trophies, by resource managers. Such age assessment is based upon age related variation in phenotypic traits such as mane size and colour, yellowing and breakage of teeth, nose pigmentation, scaring and jowl-slackness^15,30,54^. The rate at which phenotypic traits develop varies between individual lions^15,30^, and some traits are more difficult to assess in the field^55^. Nevertheless, certain age classes have distinctive phenotypic characteristics that improve the reliability of age estimates. When tested, professional hunters were most successful in accurately aging male lions of ≥ 7 and ≤ 2.9 years old, but less accurate at aging lions from 3 to 6.9 years old. Notably, ages in this latter range were more frequently over-estimated than younger or older animals. Data from Miller et al.^30^ showed that 12–35-month-old lions have a 19% risk of being aged inaccurately (see Table S4). This risk rises to 35% for 36–59-month-old lions and 49% for 60–83-month-old lions while older lions (84–107-month-old) have a 33% risk of being aged inaccurately. Incorporating misclassification of different age classes into model simulations revealed significant discrepancies between population trends for model scenarios using real and hunter estimated age, with larger declines in population for estimated age scenarios. This was particularly the case for the ≥ 5, ≥ 6 and ≥ 7-year minimum age threshold simulations of populations without a protected core where real and estimated age scenarios diverging most markedly. Taking into account age estimation errors the ‘estimated age’ ≥ 8-year scenario is, in reality, similar to a ‘real’ age ≥ 5 year scenario, whilst the ‘estimated age’ ≥ 6-year scenario was closer to a ≥ 4 year ‘real’ age scenario which is clearly unsustainable in situations when the whole population is vulnerable to hunting. This is of particular concern because the ≥ 6-year minimum age threshold has been widely implemented by resource managers^30,52^.

The findings of this study have several practical implications for lion conservation and population management. Given that very few wild lion populations are free from the negative impacts of retaliatory killing and/or poaching, we would recommend that the additive effects of these multiple additional mortality sources be explicitly considered in trophy hunting management strategies. As such hunting offtakes should be highly conservative or suspended where high levels of additional anthropogenic mortality are known to occur. In addition, we recommend that theoretical population models used to inform management decision making should account for inaccuracy in aging under field conditions to ensure hunting offtakes are sustainable. Furthermore, because error in age assessment by professional hunters could potentially have significant effects on population viability, particularly when accompanied by other anthropogenic mortality and where there is no demographic rescue from source populations, we recommend raising minimum age thresholds for trophy hunted lions to animals older than 8 years.

## Methods

### Lion population model

We developed an individual based model (IBM) in R^56^ incorporating unique features of lion social biology (territoriality, female pride and male coalition formation, non-seasonal breeding, sexually selected infanticide and male biased dispersal) (https://CRAN.R-project.org/package=pop.lion). Probabilistic rules describing demographic processes exhibited by lion populations were developed based on events occurring at individual and social group (female prides, male coalitions) level. The model was parameterized with detailed demographic, survival and mortality parameters, based on a long term demographic study of lions in Hwange National Park, Zimbabwe^18,25^, augmented with data from the published literature (see Supplementary Material, Tables S2 & S3). Each model simulation progressed at monthly time steps, with 1000 iterations and mean and quantile range between 0.025 and 0.975 calculated, for each scenario. Initial runs of the model indicated that the population reached its asymptotic state after 35 years at which point we simulated the effects of anthropogenic mortality as outlined below. Outputs of the model are described in the Supplementary Material.

### Simulation of edge effects

To simulate the source-sink dynamics of lion populations on the borders of fully protected areas due to trophy hunting, poaching and retaliatory killing (the latter two mortality sources grouped together as ‘conflict’ hereafter) we assigned a proportion of pride ranges to the ‘edge’ or ‘core’. Individuals in the ‘edge’ category were subjected to both trophy hunting and other anthropogenic mortality but not those in the ‘core’ (see Supplementary Methods for further details). We simulated a range of four scenarios each with annual hunting quotas of 18 males ≥ 6 years and 0.4% conflict mortality per month (annual mortality rate of 0.05, see below) with increasing proportions (25–100%) of prides subjected to edge effects. Dispersing individuals moved into the zone (edge or core) with the lowest lion density reflecting the source-sink movement dynamic created by the vacuum effect.

### Simulation of additive effects of trophy hunting and other anthropogenic mortality

To simulate varying levels of trophy hunting, lions were removed from vulnerable (edge) prides and coalitions at each time step. We simulated annual hunting quotas of 6, 12 and 18 male lions, equating to approximately 0.4, 0.8 and 1.2 lions/1000 km^2^ in the Hwange system. These intensities of hunting are defined as low, moderate and high offtakes and span the recommended range of sustainable off-takes of between 0.5 and 1.0 lions/1000 km^2^^57^ and approximate the range of lions hunted annually from the Hwange population^18^. Annual quotas were assigned randomly across each twelve-month period. Monthly hunting quotas were removed provided lions, of ages exceeding the defined minimum and assigned to coalitions defined as vulnerable to edge effects, were available. If not, a hunt failed, and this part of the quota was not filled.

To simulate other sources of anthropogenic mortality, at each time step a set portion of the population was removed from vulnerable edge prides, coalitions and dispersers. As snare poaching and retaliatory killing are less selective than trophy hunting this mortality source affected all age and sex demographics. We simulated zero, low, moderate, and high monthly mortalities of 0.0, 0.4, 0.8 and 1.2 percent of the population. These equate to annual mortality rates of 0.0, 0.05, 0.09 and 0.13 respectively and are plausible when compared to annual cause specific mortality rates of adult female lions in HNP (snaring-range: 0.03 ± 0.03–0.09 ± 0.08, maximum: 0.20; retaliatory killing-range: 0.02 ± 0.05–0.04 ± 0.03, maximum: 0.12^18^). The impacts of anthropogenic mortality were simulated for a population with a protected core (37% of prides), approximating the situation in HNP and a population with no protected core (100% of prides vulnerable).

### Minimum age-based thresholds for trophy hunting offtakes

We simulated minimum age thresholds (≥ 3 years up to ≥ 8 years old) by only allowing lions with ages exceeding the relevant age threshold to be hunted. We simulated six hunting management regimes with different minimum age thresholds for hunted male lions (≥ 3 to ≥ 8 years) for scenarios with 37% (25 of 40) and 100% of prides affected by edge effects under situations with low, moderate, and high conflict mortality (0.4, 0.8, 1.2% mortality per month). Simulations included age misclassification by professional hunters (see below).

### Simulation of hunter error in field age assessments

In order to assess the potential impacts of errors in field aging of lions by hunters on sustainability, we substituted ‘actual’ age of an individual in the model for ‘estimated’ age based on the frequency with which professional hunters underestimated, overestimated or correctly aged lions in each age category, after Miller et al.^30^ (Table S4). We simulated the effect of aging errors on six minimum age threshold hunting management strategies ranging from ≥ 3 to ≥ 8 years.

### Assessing the sustainability of hunting scenarios

Exploitation of animal populations is dependent on species conservation status (locally, regionally and internationally), management purpose and requirements, economic goals, public acceptance and ethical considerations all of which may vary significantly between species, regions and stakeholder groups^58,59^. For example, resource managers may accept depletion of commercially exploited populations to well below their natural carrying capacity (or indeed aim to deplete or eradicate populations of invasive or pest species), whilst considering even small declines in native populations in fully protected areas, particularly of keystone species such as predators, to have unacceptable impacts on ecosystem function or species persistence. Such views may vary widely and differ markedly for different species and between local and international contexts, such that a single definition of sustainable exploitation is impossible. For the purposes of quantifying the responses of simulated lion populations to model scenarios, we considered declines from initial populations of more than 10% to be inadvisable, with the rationale that such declines in a globally vulnerable apex predator would be significant cause for concern. Nevertheless, resource managers might accept larger initial population declines provided populations remained stable (λ =  > 1) thereafter. We evaluate model scenario outcomes according to both criteria but note that these are necessarily arbitrary and may not be generally applicable to real-world lion population management scenarios.

## Supplementary Information

Supplementary Information.

## Data Availability

All data used in the model are provided in the manuscript and supplementary material or readily available. Model code is provided in an open access repository (https://CRAN.R-project.org/package=pop.lion).
