# Supplementary material for: Anthropogenic edge effects and aging errors by hunters can affect the sustainability of lion trophy hunting
Source: Sci Rep. 2023 Jan 12;13:95. doi: 10.1038/s41598-022-25020-9 (PMC9837042; doi:10.1038/s41598-022-25020-9)
Supplement: Supplementary file 1 — Supplementary Information. [file 41598_2022_25020_MOESM1_ESM.docx]

**Anthropogenic edge effects and aging errors by hunters can affect the sustainability of lion trophy hunting.**

Andrew J. Loveridge, Matthew Wijers, Roseline Mandisodza-Chikerema, David W. Macdonald, Guillaume Chapron.

**Supplementary Material**

**Supplementary methods**

Lion population model

We developed a lion-specific individual based model, re-using the code foundation of a previous model for European wolves (*Canis lupus*), see [1] for details, that models events at individual, pride and coalition levels and formalizes them into probabilistic rules. The purpose of the model is to describe the dynamic and structure of a lion population. The model is developed in the language C [2] and uses functions from the Glib library [3]. It is made available as a multi-platform and open source R package ‘pop.lion’. (<https://CRAN.R-project.org/package=pop.lion>). We follow the standard Overview, Design concepts, and Details (ODD) protocol to describe the model [4].

*State variable and scales*

The model comprises three hierarchical scales: individuals, prides and coalitions, and population (Figure S1). **Individuals** are characterized by the following state variables: identity number, alive status, sex, stage (resident or vagrant/disperser), age (in months), dispersal status (natal or dispersed), mating status (mated or not mated), time since mating (in months), hunter estimated age (in months) and pointers to their mother, their litter and to the pride or coalition to which the individual may belong. Age classes are yearly (from 0-15) and are used to set individual survival rates. A **pride** is defined as a group of related adult lion females with their male and female offspring, with related meaning that they were born in the same pride. A **coalition** is defined as a group of (related or unrelated) adult male lions which may acquire one or several prides for reproduction. Both prides and coalitions are characterized by the following state variables: identity number, stage (resident or vagrant/disperser), list of individuals, time in months the pride or coalition has been resident or vagrant and pointers to their associated pride (for a coalition) or coalition (for a pride). Prides also include a spatial status depending on whether the pride is on the edge of a protected areas and vulnerable to edge effects (edge) or in the fully protected core of a protected area (core). The **population** consists of all individuals, prides and coalitions. The population also keeps track of the cumulative number of individuals that are alive, the number of resident prides, the number of prides that are at the edge, the number of coalitions and the number of resident coalitions. We did not model immigration or emigration into or out of the discretely modelled population. However, emigration is implicitly included as sub-adults not finding vacant territories within a defined period (6 months) are eliminated from the model. Immigration into the population occurs only sporadically [5].


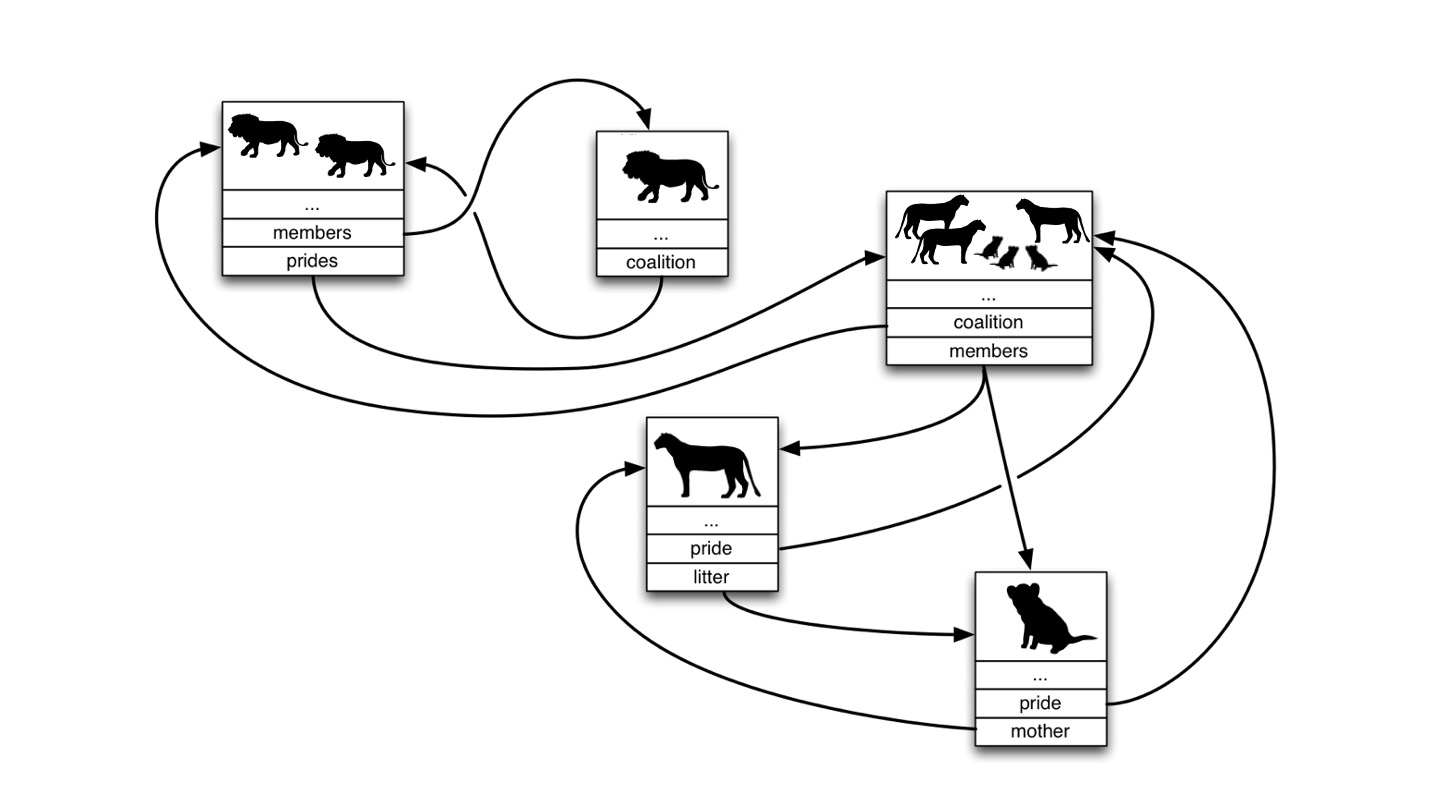


Figure S1: Schematic representation of object types in the lion population model with connections between objects.

*Process overview and scheduling*

The model proceeds in monthly time steps. Within each month, the following sequence of events takes place in this order:

1. Individuals, prides and coalitions age
2. Individuals survive or die
3. Individuals disperse from prides and coalitions
4. Prides settle
5. Coalitions split
6. Coalitions merge
7. Coalitions fight
8. Coalitions mate with prides
9. Prides reproduce
10. Collection of statistics

The details of each event are provided further below.

*Design concept*

**Emergence and interactions:** The population dynamic emerges from events occurring at the individual level according to biological rules and parameters. The model includes dynamic feedback on pride settlement and cub mortality. New prides are assigned an edge status depending on the outcome of a Bernoulli trial based on the population density relative to carrying capacity in each zone (prides are more likely to settle in the zone with the lower population density). Vagrant/ dispersing males and females are assigned either edge or core status according to the outcome of a Bernoulli trial based on the proportion of prides at the edge when the population is at carrying capacity (a proxy for the edge:core area ratio). Cub mortality due to starvation (years 0-1 and 1-2) is dependent on the outcome of a Bernoulli trial based on the population density relative to carrying capacity multiplied by a fixed factor (0.03 for year 1 and 0.01 for year 2) to simulate the positive relationship between population density and cub mortality that is evident from comparisons made between the Serengeti and Hwange National Parks [17].

**Observation:** A model simulation returns a list consisting of 1) an array with dimension (time, statistics, runs) where time is the simulation length in months; statistics are demographic data including number of lions, number and size of settled and transient prides and coalitions in both the edge and the core, sex and age structure, number of litters and pride takeovers and runs are single stochastic trajectories; 2) an array with dimension (cumulative total number of individuals, statistics) where statistics indicate for each individual the time it was born, dispersed, settled, bred for the first time and died (from an anthropogenic causes, infanticide, a fight, starvation, or other natural cause).

*Initialization*

Each simulation starts with the same initial population, defined by number of lions arranged in prides and coalitions. The initial population size and structure in the simulations are taken from observed data on Hwange (Table S1 and S2).

- Initial number of prides / coalitions
- Number of individuals in initial prides
- Number of individuals in initial coalitions

Carrying capacity (K_individuals), which is based on empirical estimates, remains fixed for the duration of the simulation. The model therefore does not consider changes in this factor over time which may result from fluctuating prey abundance.

Table S1. Input parameters for a simulation

| **Parameter** | **Value** |
| --- | --- |
| K individuals | 500 |
| K prides | 40 |
| K coalitions | 40 |
| K edged | variable |
| Initial population (prides) | 4 |
| Number of runs | 1000 |
| Conflict age threshold (months) | 0 |
| Conflict mortality (%) | variable |
| Hunting age threshold (months) | variable |
| Hunting mortality (monthly quota) | variable |
| Hunter error | 0/1 |

*Input*

The model requires information on survival, dispersal, settling and reproduction (Table S2) to serve as parameters for Monte Carlo simulations.

Model inputs are derived from published data available in the literature [6-15] and from a long term, intensively monitored population in western Zimbabwe in a study area covering approximately 7000km^2^ in northern and eastern Hwange National Park (HNP, 14 500km^2^), and surrounds from 1999-2019. In total the life histories of 920 individuals, in 46 prides and 32 singletons and coalitions of males were documented through detailed observations of study prides, augmented by collated professional tourist guide photographs and sightings [16-24]. Data included pride compositions and sizes, coalition sizes, tenure and pride relationships, breeding, birth dates (or estimated ages where these were unknown), dispersal events, deaths, and mortality sources. This population forms part of the greater Okavango-Hwange population within the Kavango-Zambezi (KAZA) Transfrontier Conservation Area [25]. The protected area is surrounded by safari areas, forestry and private lands and communal lands, all of which receive annual quotas to hunt lions, but also where lions are killed in wire snares and by livestock farmers (see Loveridge et al 2016 [18] for a full site description and details of hunting quotas and anthropogenic mortalities). The HNP population is exposed to multiple sources of anthropogenic mortality, including trophy hunting (of male lions, but historically also females), legal problem animal control (PAC) by wildlife authorities, illegal retaliatory killing by livestock owners, wire snare poaching by bush meat hunters and lethal collisions with vehicles (trains and large trucks) [19,26]. Survival and cause-specific mortality rates were calculated for each age class (yearly from 0-15) using cumulative incidence functions for competing risk events [27] and interpolated for ages where we did not have sufficient data. Baseline survival rates used in the model included only natural sources of mortality (disease, mortalities due to interactions with dangerous prey or other predators; Table S3). We carried out a parameter elasticity analysis for parameters relating to fights, fecundity, starvation, and baseline survival by varying each parameter one at a time while keeping other parameters constant (Figure S8 and S9). We used growth rate as the dependent variable which was calculated between year 10 and year 20 of simulations with 1000 iterations.

Table S2. Demographic model parameters.

| **Parameter** | **Value** | **Source** |
| --- | --- | --- |
| Sex ratio | 0.5 | Hwange demographic data |
| Mean number adult females per pride | 4 | Hwange demographic data |
| Mean age of adult females | 72 months | Hwange demographic data |
| Number of cubs per adult female | 0.34 | Hwange demographic data |
| Number of yearlings per adult female | 0.74 | Hwange demographic data |
| Number sub-adults (1-2) per adult female | 0.74 | Hwange demographic data |
| Number sub-adults (3-4) per adult female | 0.74 | Hwange demographic data |
| Mean coalition size | 2.6 | Hwange demographic data |
| Mean age of adult males | 72 months | Hwange demographic data |
| Maximum coalition size | 4 | Hwange demographic data |
| Age at first reproduction | 48 months | Hwange demographic data |
| Age that cubs die without mother | 12 months | Author expert opinion based on field experience |
| Age that cubs die without surrogate | 24 months | Author expert opinion based on field experience |
| Age of surrogate mother | 48 months | Author expert opinion based on field experience |
| Age to survive infanticide | 32 months | Elliot et al. 2014 [21] |
| Age female dispersal begins | 28 months | Elliot et al. 2014 [21] |
| Age female dispersal ends | 45 months | Elliot et al. 2014 [21] |
| Age female dispersal triggered by takeover | 20 months | Elliot et al. 2014 [21]; Hanby & Bygott 1987 [7] |
| Age female triggered dispersal ends | 30 months | Elliot et al. 2014 [21]; Hanby & Bygott 1987 [7] |
| Age male dispersal begins | 28 months | Elliot et al. 2014 [21] |
| Age male dispersal ends | 45 months | Elliot et al. 2014 [21] |
| Maximum pride size | 10 | Pusey & Packer 1987 [21]; VanderWaal et al. 2009 [13] |
| Maximum time pride can be vagrant | 6 months | Whitman et al. 2004 [10] use 0 |
| Maximum time coalition can be vagrant | 6 months | Whitman et al. 2004 [10] use 0 |
| Probability of male mortality from a fight | 0.15 | Grinnell et al. 1995 [8]; West et al. 2006 [9] |
| Probability of female mort. from a fight | 0.15 | West et al. 2006 [9] |
| Resident advantage factor in a fight | 1.5 | Starfield et al. 1981 [15]; Whitman et al. 2004 [10] |
| Mating success | 0.25 | Lehmann et al. 2008 [14] |
| Monthly probability of starvation for year 1 | 0.03 | Schaller 1972 [6] (28% cub deaths due to starvation.) |
| Monthly probability of starvation for year 2 | 0.01 | Schaller 1972 [6] |

Table S3. Age-specific baseline survival rates (annual) based on long-term monitoring data from Hwange National Park. These survival rates are based on sources of mortality not included in the model mechanics, such as disease, being killed by dangerous prey, and vehicle impacts.

| **Age (years)** | **Survival Rate** |
| --- | --- |
| 0-1 | 0.97 |
| 1-2 | 0.98 |
| 2-3 | 0.99 |
| 3-4 | 0.99 |
| 4-5 | 0.99 |
| 5-6 | 0.99 |
| 6-7 | 0.99 |
| 7-8 | 0.99 |
| 8-9 | 0.98 |
| 9-10 | 0.96 |
| 10-11 | 0.94 |
| 11-12 | 0.92 |
| 12-13 | 0.90 |
| 13-14 | 0.87 |
| 14-15 | 0.83 |

*Sub-models*

1. Individual, pride and coalition ageing

The age of individuals, prides and coalitions is incremented by 1 month at each step. Each individual is also assigned a hunter-estimated age based on data from Miller et al. (2016) [28] as shown in Table S4.

Table S4. Hunter-estimated age based on Miller et al. (2016) [28].

| **True Age (months)** | **Estimated Age (months)** | **% Classifications** |
| --- | --- | --- |
| 12-35 | 12-35 | 81 |
|  | 36-59 | 17 |
|  | 60-83 | 2 |
| 36-59 | 12-35 | 12 |
|  | 36-59 | 65 |
|  | 60-83 | 18 |
|  | 84-107 | 5 |
| 60-83 | 12-35 | 2 |
|  | 36-59 | 14 |
|  | 60-83 | 51 |
|  | 84-107 | 32 |
| 84-107 | 36-59 | 5 |
|  | 60-83 | 28 |
|  | 84-107 | 67 |

1. Individual survival

Every month, a proportion of individuals die in one of the following ways:

- Baseline mortality which incorporates causes that are not explicitly included in the model (e.g., disease). An individual’s survival is dependent on the outcome of a Bernoulli trial based on the age-specific mortality rate (Table S3).
- Senescence: all individuals > 15 years old will die
- Cubs younger than 12 months always die if their mother dies. Cubs older than 12 months survive if their mother dies as long as there is a mother at least 4-year-old in the same pride that can act as a surrogate. At age 24 months, cub survival is not influenced by their mother’s death.
- Cubs can die of starvation; depending on the outcome of a Bernoulli trial based on the population density relative to carrying capacity multiplied by the probability of starvation for the relevant year group.
- Individuals can die from hunting or conflict if they meet specific criteria:
  - Sex (e.g., only males are vulnerable to hunting)
  - Age, which needs to be equal to or greater than the age threshold.
  - In the edge zone and therefore vulnerable to hunting or conflict:
    - A resident coalition’s edge status depends on that of its prides. If more than half the number of prides held by a coalition are at the edge, its edge status is dependent on the outcome of a Bernoulli trial based on the proportion of prides at the edge. Otherwise, the coalition is assigned core status.
    - The edge status of disperser prides and coalitions is dependent on the outcome of a Bernoulli trial based on the size of the edge carrying capacity relative to the core carrying capacity (as a proxy for size).
- Cubs can die from infanticide as described in point 3 below.

1. Individual dispersal from prides and coalitions

All females aged 28-45 months will leave their natal pride and form a vagrant or dispersing pride when the number of females in the pride that are older than 28 months exceeds 10 individuals. All females aged 20-30 months will also disperse when a new male joins the pride (hereafter termed “triggered dispersal”). Females that have not dispersed before 45 months will remain in their natal pride. Subadult males disperse with subadult females as above and also when the age of oldest subadult male is 45 months. All sub-adult males eventually disperse and form a vagrant or dispersing coalition and never remain with their natal pride.

If a new adult male joins a pride, infanticide occurs. This happens following a takeover when coalitions fight, or when a new coalition joins a pride without an existing coalition. The probability of a cub being killed is dependent on age and sex [7,12,21]. When the cub sex is female, this individual dies according to a Bernoulli trial based on the age-specific probabilities in Figure S2.


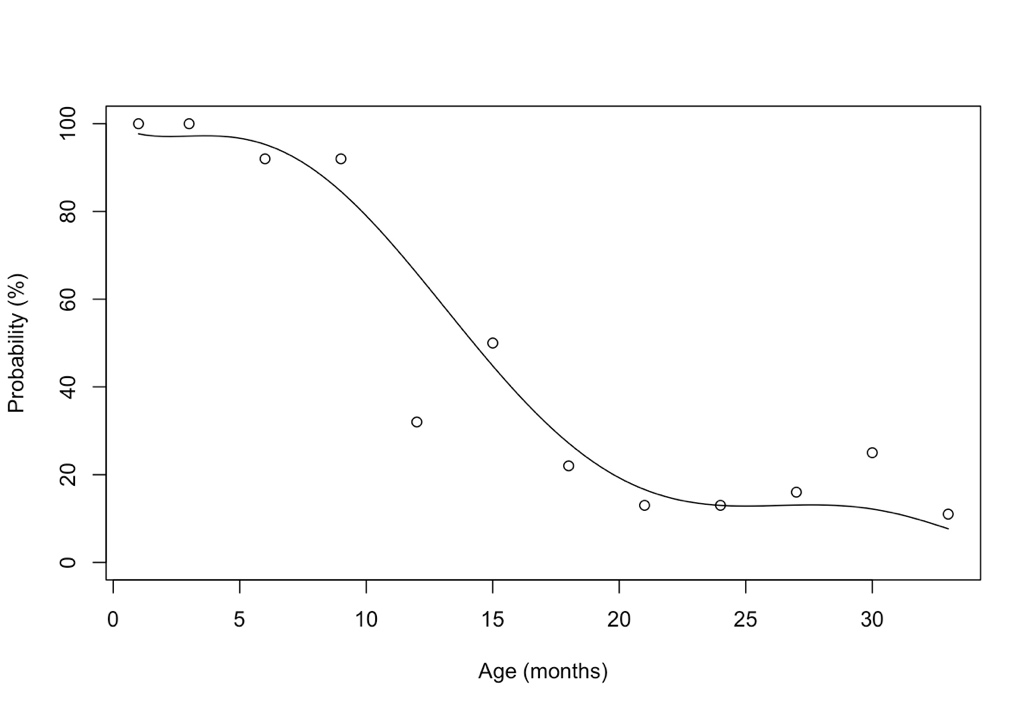


Figure S2. Probability of a female cub being killed following a pride takeover. Probability is based on data from Pusey and Packer (1987)[12]. We fit a trendline where the probability of being killed = 2.9539E-06x^6^ - 0.000334047x^5^ + 0.0137274757x^4^ - 0.2391477426x^3^ + 1.4663687561x^2^ - 3.5297032314x + 100. If the cub sex is male, the individual will die if it is younger than the age at which lions survive infanticide (32 months).

1. Pride settlement

Every month, every dispersing pride will attempt to settle if there is a vacant territory. For each dispersing pride, settlement depends on the outcome of a Bernoulli trial based on the number of ranges available relative to the total number of ranges (K prides). If a dispersing pride successfully settles, it is assigned to the spatial zone (edge or core) with the lowest pride density. If the landscape is saturated with prides, dispersing prides remain as dispersers until a territory becomes vacant but can only do so up until 6 months after which they are removed from the population to simulate disperser death or emigration. Coalitions do not settle directly but always through association with resident prides. Dispersing coalitions are removed from the model after 6 months if unable to settle during this time period.

1. Coalition splitting

Coalitions cannot be larger than 4 males (which is the maximum stable coalition size observed in HNP). When a coalition has more than 4 individuals, the model removes 2 or 3 individuals (with equal probability) from the original coalition and forms a new dispersing coalition.

1. Coalition merging

Dispersing singleton males attempt to form coalitions with other males. Every month, each singleton dispersing male will try to join 1 other single dispersing male to form a dispersing coalition of 2 males.

1. Coalition fighting

Every month a dispersing coalition will challenge one resident coalition to take over one of its prides. The outcome of the challenge is based on each coalitions’ size and age. Success of the challenge is determined using the following method:

- List males in coalitions and aged 2-15 years.
- Assign an age-based handicap score (H-score) to each individual in each coalition with 6 being prime age (Table S5; handicap increases below and above 6).
- Square the handicap score because the relationship between age and strength is not linear.
- Convert handicap to strength score by subtracting from 101 so that maximum strength score at 6 = 100.
- Multiply the dispersing coalition score by resident advantage (1.5) to get resident coalition score. This is higher because all things being equal, the resident coalition should still have an advantage. The logic behind the Whitman et al. 2004 [10] fight matrix assumes that 3 x 6-year-old dispersers are an even match for 2 x 6-year-old resident males.

Table S5. Summary of H-scores allocated to each age group

| **Age (years)** | **2** | **3** | **4** | **5** | **6** | **7** | **8** | **9** | **10** | **11** | **12** | **13** | **14** | **15** |
| --- | --- | --- | --- | --- | --- | --- | --- | --- | --- | --- | --- | --- | --- | --- |
| H score | 10 | 7.75 | 5.5 | 3.25 | 1 | 2 | 3 | 4 | 5 | 6 | 7 | 8 | 9 | 10 |
| H score^2^ | 100 | 60.1 | 30.3 | 10.6 | 1 | 4 | 9 | 16 | 25 | 36 | 49 | 64 | 81 | 100 |
| DC strength | 1 | 40.9 | 70.7 | 90.4 | 100 | 97 | 92 | 85 | 76 | 65 | 52 | 37 | 20 | 1 |
| RC strength | 1.5 | 61.4 | 106.1 | 135.6 | 150 | 146 | 138 | 128 | 114 | 97.5 | 78 | 55.5 | 30 | 1.5 |

- Calculate the chance of resident coalition winning as: $\left( \frac{Total strength RC}{Total strength DC} \right)^{{(RC size -DC size)}^{2}+2}$

Where RC is the resident coalition and DC is the dispersing coalition. Examples are provided below.

- If the probability of the resident coalition winning the fight is between 0.15 and 0.70 then each member of the losing coalition can die following a fight (resident and dispersing coalitions would likely engage in a physical encounter if they had a reasonable chance of winning). An individual will die following a Bernoulli trial with probability 0.15.

If the resident coalition loses the fight, infanticide occurs, mothers with litters in the pride have 0.15 probability of being killed and triggered dispersal occurs. If the resident coalition had only one pride, it will then become vagrant, otherwise it will remain resident with its other prides (see examples in Appendix).

1. Coalitions join prides

Every month, vagrant and resident coalitions search for available prides (without a current coalition) to join. For each coalition, successfully finding another pride follows a Bernoulli law based on number of available prides relative to K prides and the current number of existing prides held by the coalition as follows:

Probability = $\frac{\left( \frac{N prides available}{K prides} \right)}{\left( N current prides \right)^{2}+ 1}$

When a coalition successfully joins a pride, infanticide occurs, mothers with litters in the pride have 0.15 probability of being killed and triggered dispersal occurs.

1. Pride reproduction

Reproduction occurs year-round. As soon as a female reaches the age of first reproduction (48 months), they mate with a member of the pride’s coalition. Conception follows a Bernoulli law based on the probability of mating success (0.25). If mating is successful, the female will give birth in the fourth month following conception. The size of the litter is determined by a tabulated probability distribution shown in Table S6 (based on litter size from HNP) with each cub having a 0.5 probability of being male or female:

Table S6. Probability distribution of lion litter size in HNP.

| **Number of cubs** | **Probability** |
| --- | --- |
| 1 | 0.12 |
| 2 | 0.30 |
| 3 | 0.35 |
| 4 | 0.19 |
| 5 | 0.04 |

A female cannot breed until its cubs disperse and leave the pride, or, for cubs that stay in the pride, until they become independent at 48 months. If all cubs die (either naturally or by infanticide), the female can become pregnant again after 1 month.

1. Collecting statistics

The model calculates the following simulation statistics for each month:

- Number of individuals
- Number of prides
- Number of coalitions
- Number of resident coalitions
- Number of vagrant/disperser coalitions
- Number of resident prides
- Number of vagrant/disperser prides
- Resident coalition size
- Vagrant/disperser coalition size
- Resident pride size
- Vagrant/disperser pride size
- Number of females
- Number of males
- Number of pride takeovers
- Number of litters born
- Mean age of all individuals
- Number of adult males
- Number of adult females
- Number of prides (edge)
- Number of prides (core)
- Pride size (edge)
- Pride size (core)
- Number of females per pride (core)
- Number of cubs per pride (core)
- Number of females per pride (edge)
- Number of cubs per pride (edge)
- Number of individuals (core)
- Number of individuals (edge)
- Percentage of failed hunts
- Number of prides per coalition
- Inter-birth interval
- Number of adult males (edge)
- Number of adult males (core)

**Supplementary Results**

Figure S3


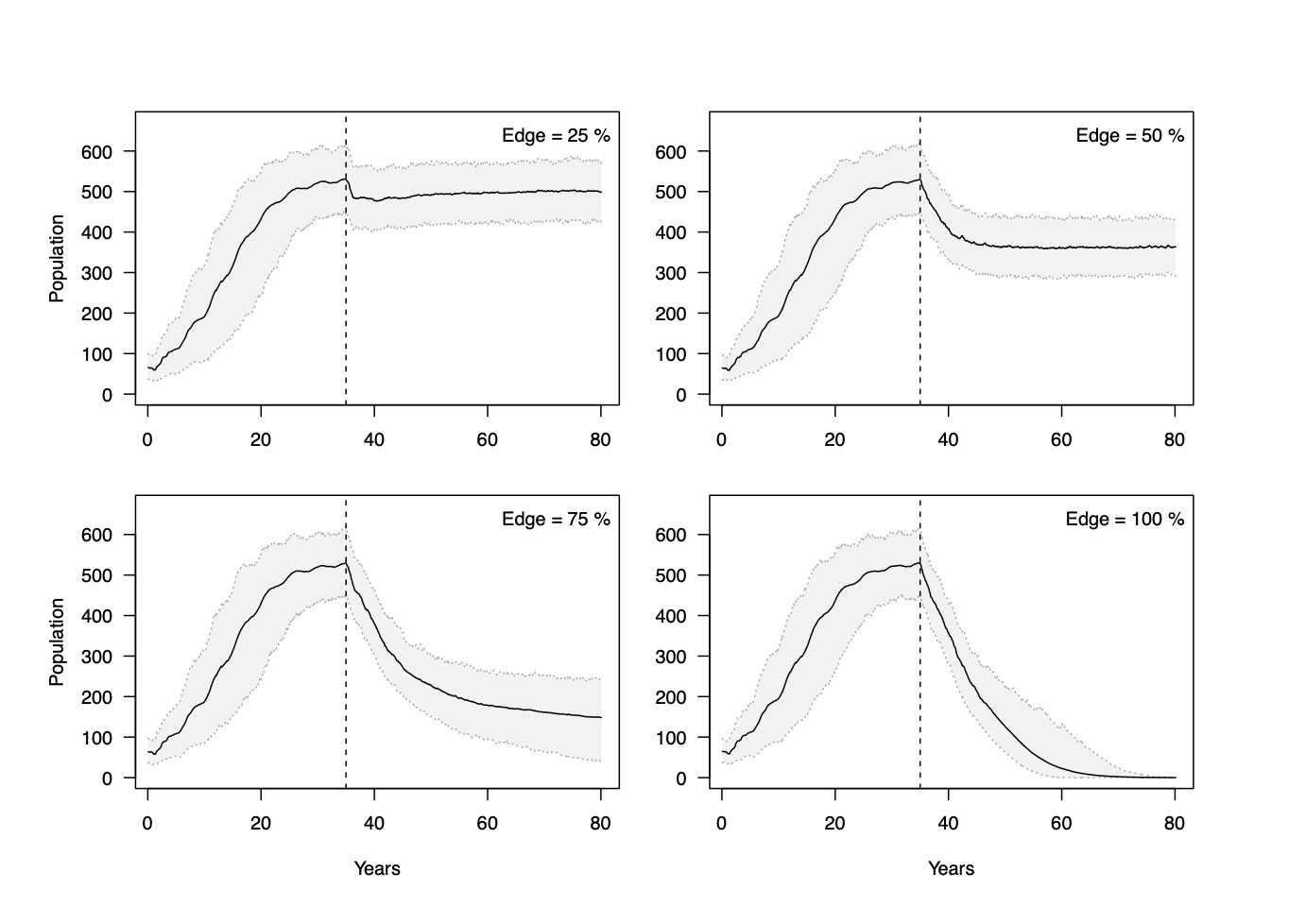


Figure S3. Effect of hunting (annual hunting quota = 18 males ≥ 4 yrs) and conflict (0.4% monthly mortality rate) on lion population persistence with varying edge effects. The continuous black line represents the mean and the light grey area the 95% confidence interval. The vertical dotted line marks the point at which test scenarios were implemented after the population had reached its asymptotic state.

Figure S4


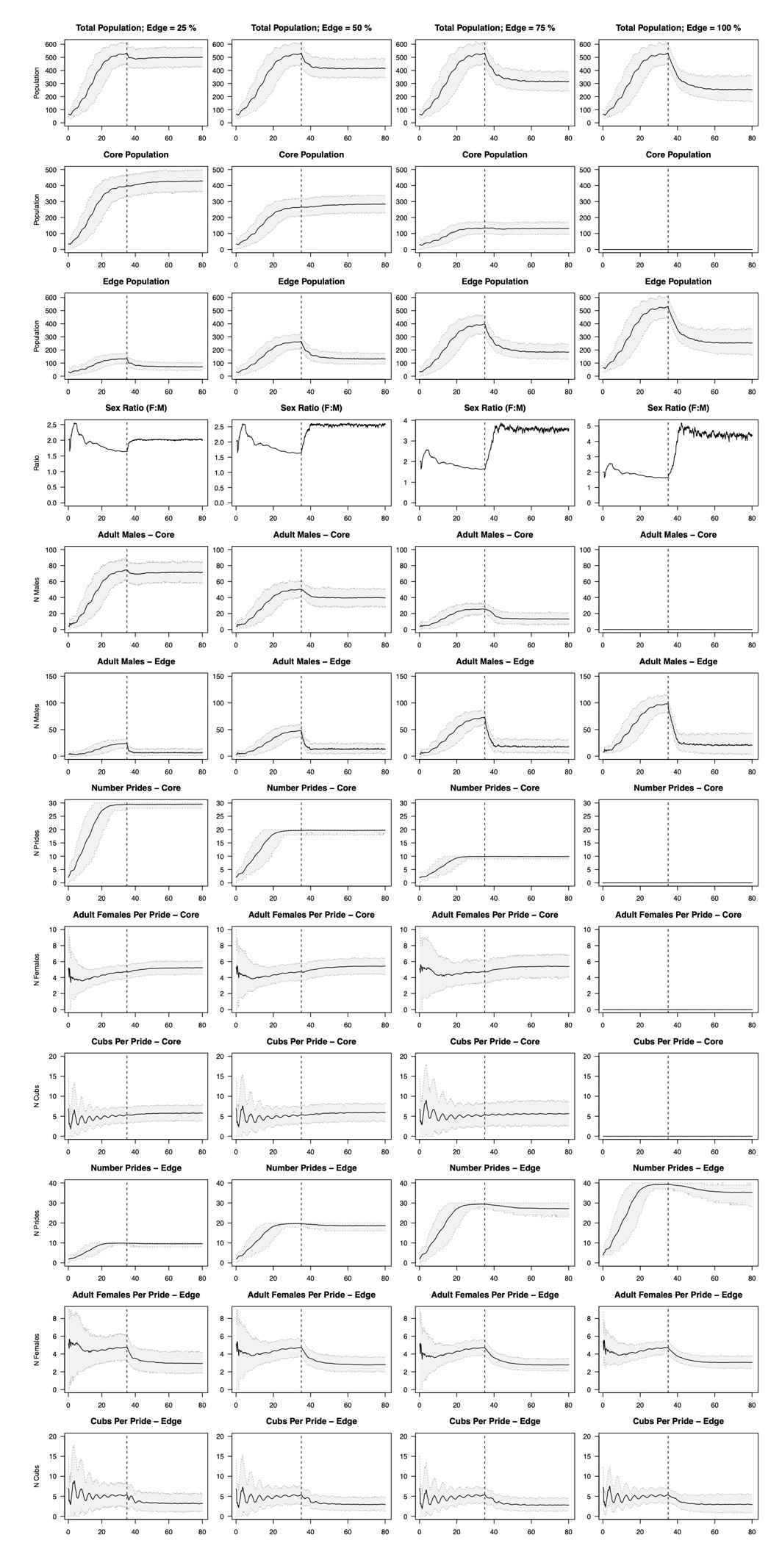


Figure S4. Outputs of model simulations showing changing population demographics in edge and core prides with increasing proportions of prides in the population impacted by edge effects (columns left to right). Monthly conflict mortality rates of 0.4% and annual hunting quotas of 18 males ≥ 6 years were used for all simulations. The continuous black line represents the mean and the light grey area the 95% confidence interval. The vertical dotted line marks the point at which test scenarios were implemented after the population had reached its asymptotic state.

Figure S5 a&b


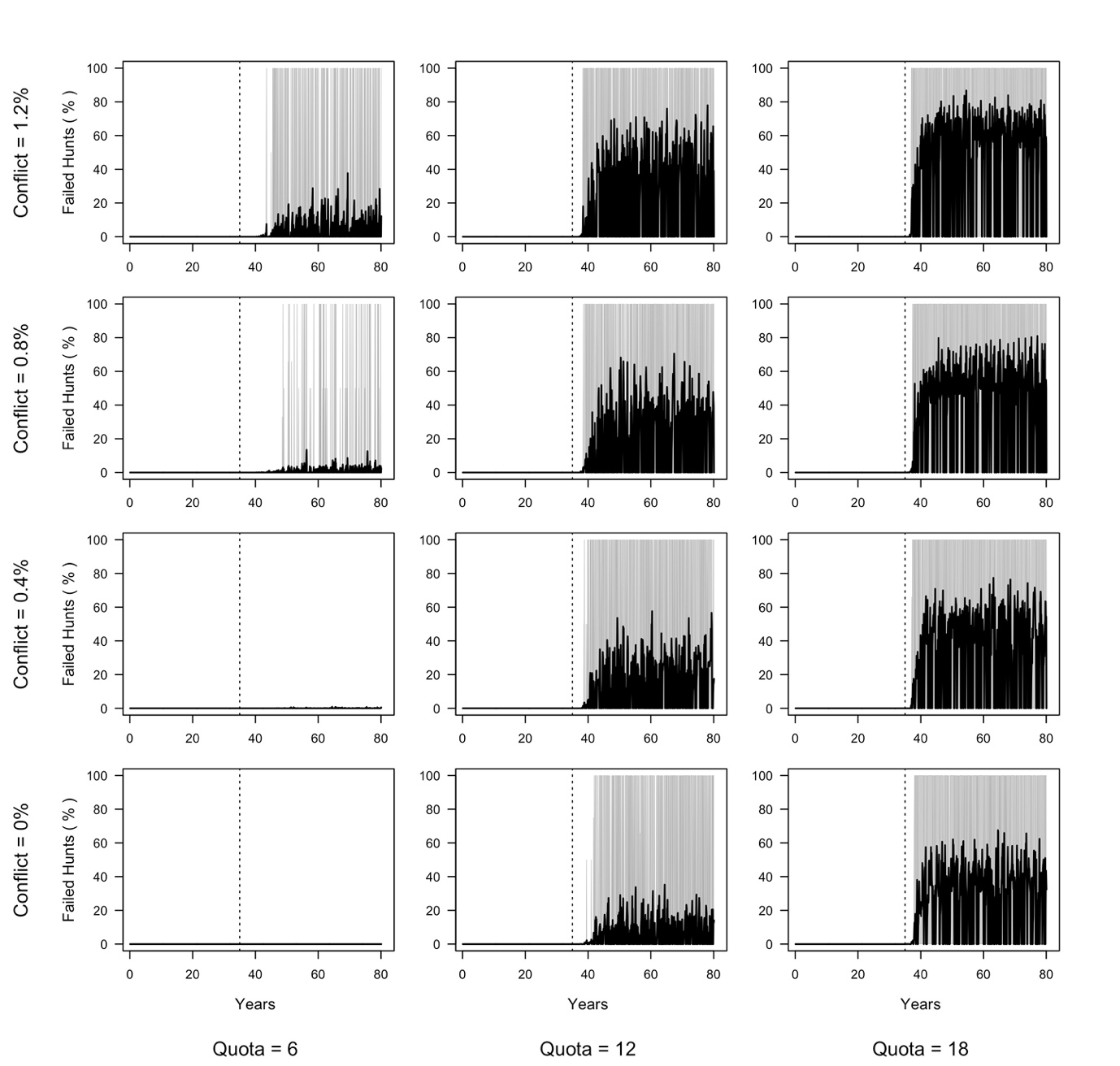


Figure S5a. Percentage of failed hunts in scenarios with increasing levels of conflict and hunting mortality. 25 of 40 prides vulnerable to edge effects, hunting offtakes restricted to males ≥6 years. The continuous black line represents the mean and the light grey area the 95% confidence interval. The vertical dotted line marks the point at which test scenarios were implemented after the population had reached its asymptotic state.


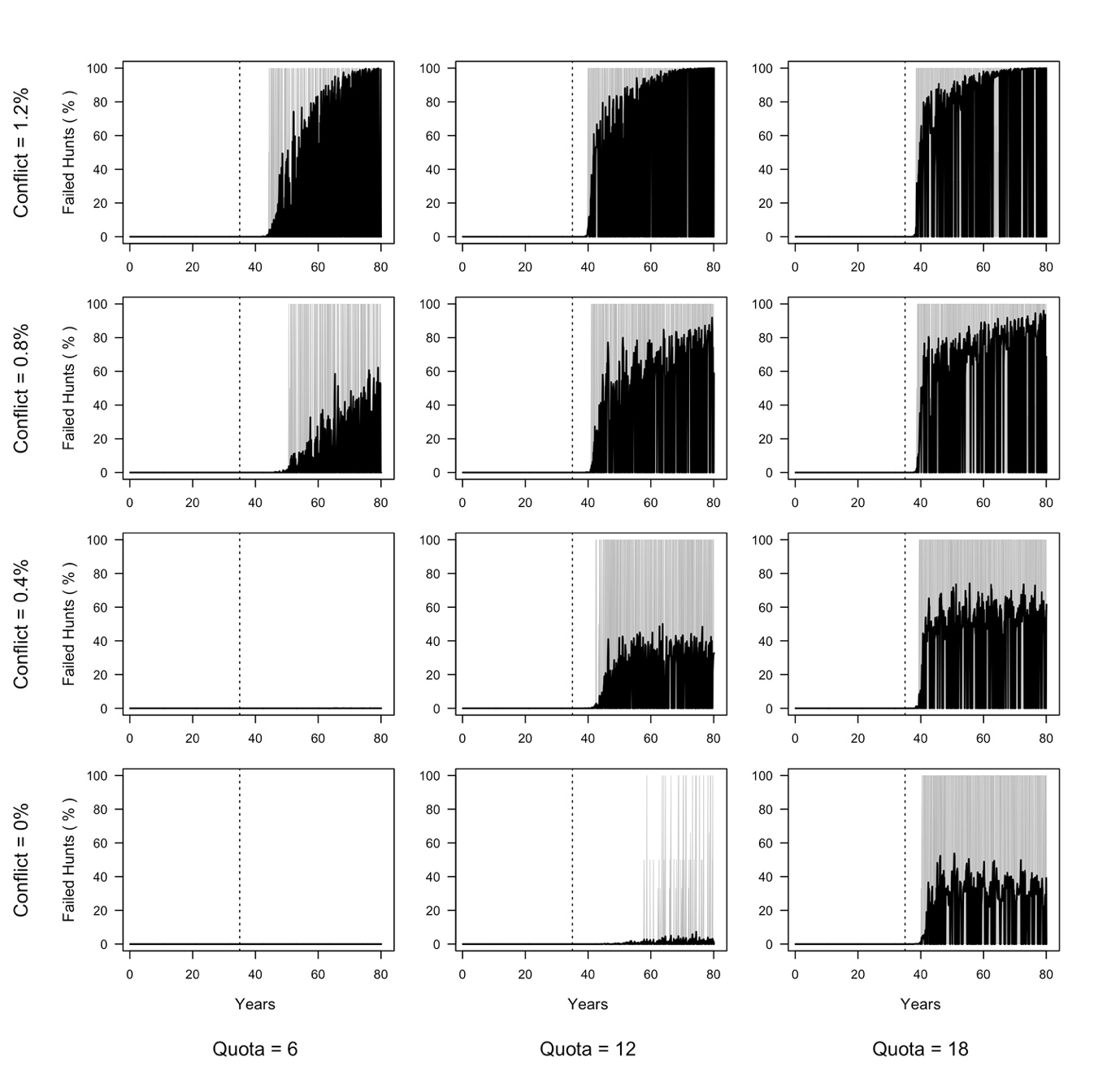


Figure S5b. Percentage of failed hunts in scenarios with increasing levels of conflict and hunting mortality. Entire population vulnerable to edge effects, hunting offtakes restricted to males ≥6 years. The continuous black line represents the mean and the light grey area the 95% confidence interval. The vertical dotted line marks the point at which test scenarios were implemented after the population had reached its asymptotic state.

Table S7

a)

| Hunting quota (per 1000km^2^) | Edge effect 25 of 40 prides (37% protected source) | | | |
| --- | --- | --- | --- | --- |
| 1.2 | → -19% | → -32% | → -40% | → -47% |
| 0.8 | → -14% | → -30% | → -40% | → -46% |
| 0.4 | → -4% | → -23% | → -36% | → -44% |
| 0 | → <-1% | → -17% | → -32% | → -40% |
|  | 0 | 0.4 | 0.8 | 1.2 |
|  | Conflict (monthly %) | | | |

b)

| Hunting quota (per 1000km^2^) | Edge effect 40 of 40 prides (no protected source) | | | |
| --- | --- | --- | --- | --- |
| 1.2 | → -26% | →-52% | ↓ -88% | ↓ -100% |
| 0.8 | → -16% | → -48% | ↓ -87% | ↓ -100% |
| 0.4 | → -5% | → -38% | ↓ -82% | ↓ -100% |
| 0 | → <-1% | → -30% | ↓ -61% | ↓ -92% |
|  | 0 | 0.4 | 0.8 | 1.2 |
|  | Conflict (monthly %) | | | |

Table S7. Percentage decline in populations from start to end of simulation (45 years) and slope (indicated by symbols) of trajectory in final 21 years (3 lion generations) of the simulation. Scenario a) 63% of prides subjected to edge effects (hunting and conflict), the remainder protected. Scenario b) all prides in simulation subjected to hunting and conflict. Scenario a) approximates a fully protected area (such as a national park) where trophy hunting, poaching and human wildlife conflict on the boundary impact survival of lions in prides close to the boundary, exerting an edge effect on these prides. Scenario b) simulates hunting areas or concessions with varying levels of investment in regulation of poaching or human wildlife conflict.

Symbols indicate stable (→, < -10.0 % slope or increasing) or declining (↓, > -10.0 % slope) populations over final 21 years of simulation. Grey shaded (≤ 10% decline in population size over 45 years) and diagonal hashed cells (≤ 30% decline in population size over 45 years) indicate management scenarios that managers might consider acceptable for management strategies for different types of protected land (≤ 30% decline and stable for consumptive use areas and ≤ 10% decline and stable for fully protected areas).

Figure S6

­


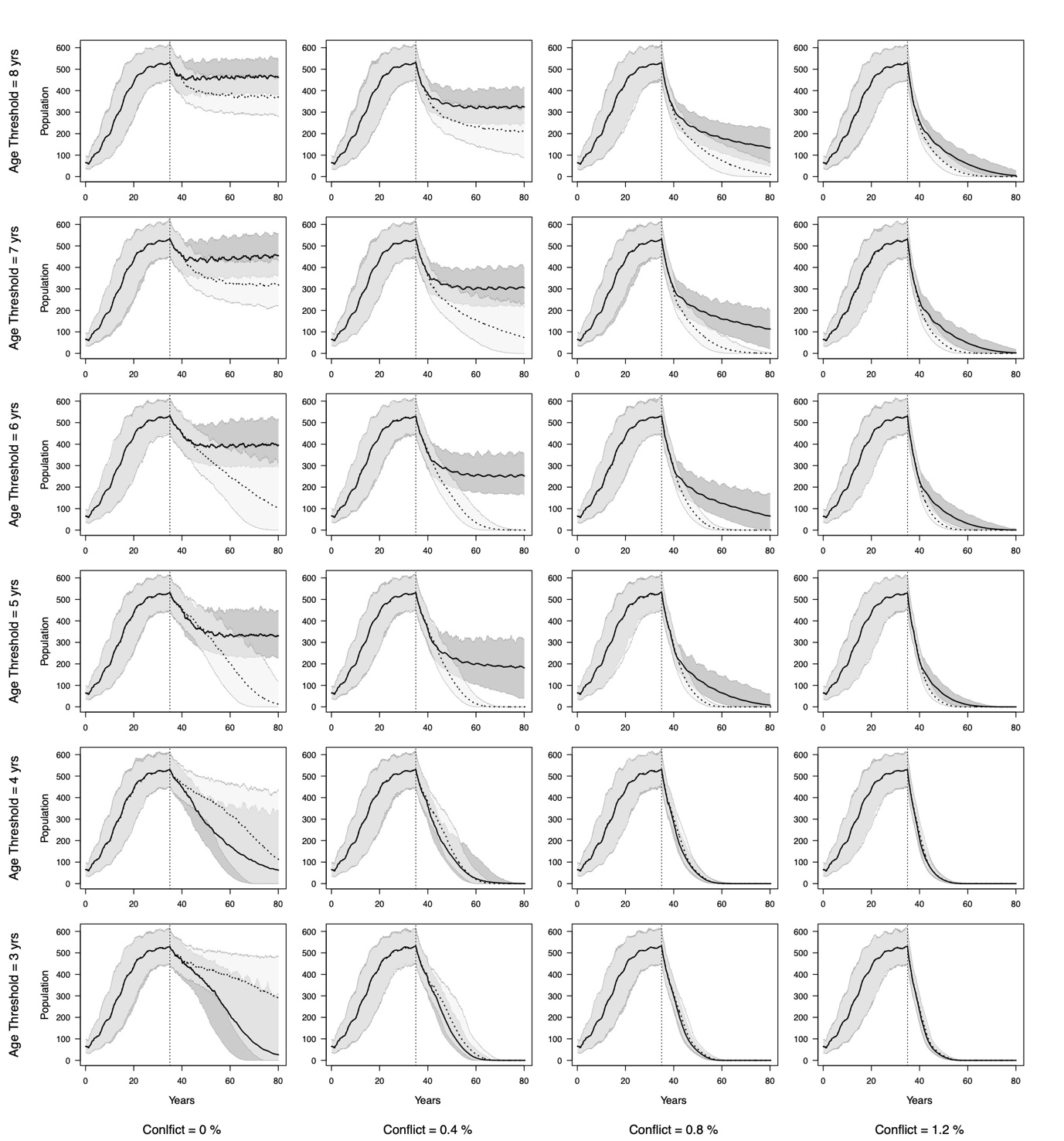


Figure S6a: Simulations of hunting male lions minimum age thresholds from ≥3 years to ≥8 years, with an annual hunting quota of 18 male lions (~1.2 lions/ 1000km^2^) and with all 40 prides in the population exposed to hunting and conflict mortalities (0.4, 0.8 and 1.2% per month). Curves with solid lines denote scenarios where ‘real’ ages of hunted lions were derived from the model and dotted lines incorporate age specific errors in age estimation by hunters. The black lines represent the mean and the light grey areas the 95% confidence interval of simulations. The vertical dotted line marks the point at which test scenarios were implemented after the population had reached its asymptotic state.


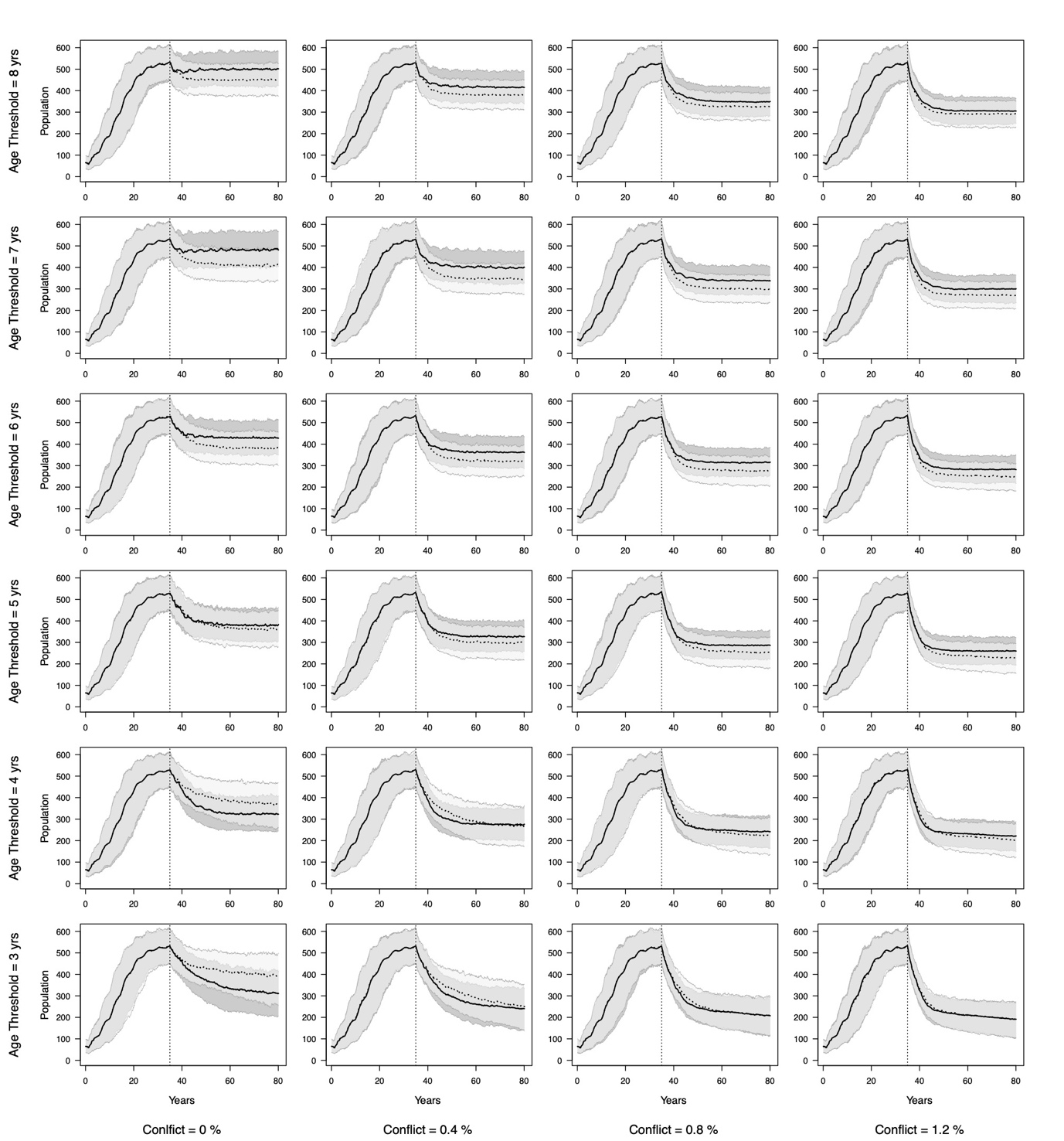


Figure S6b: Simulations of hunting male lions minimum age thresholds from ≥3 years to ≥8 years, with an annual hunting quota of 18 male lions (~1.2 lions/ 1000km2) and 25 of 40 (37%) prides exposed to edge effects (including hunting and conflict mortalities of 0.4, 0.8 and 1.2%). Curves with solid lines denote scenarios where ‘real’ ages of hunted lions were derived from the model and dotted lines incorporate age specific errors in age estimation by hunters. The black lines represent the mean and the light grey areas the 95% confidence interval of simulations. The vertical dotted line marks the point at which test scenarios were implemented after the population had reached its asymptotic state.

Figure S7


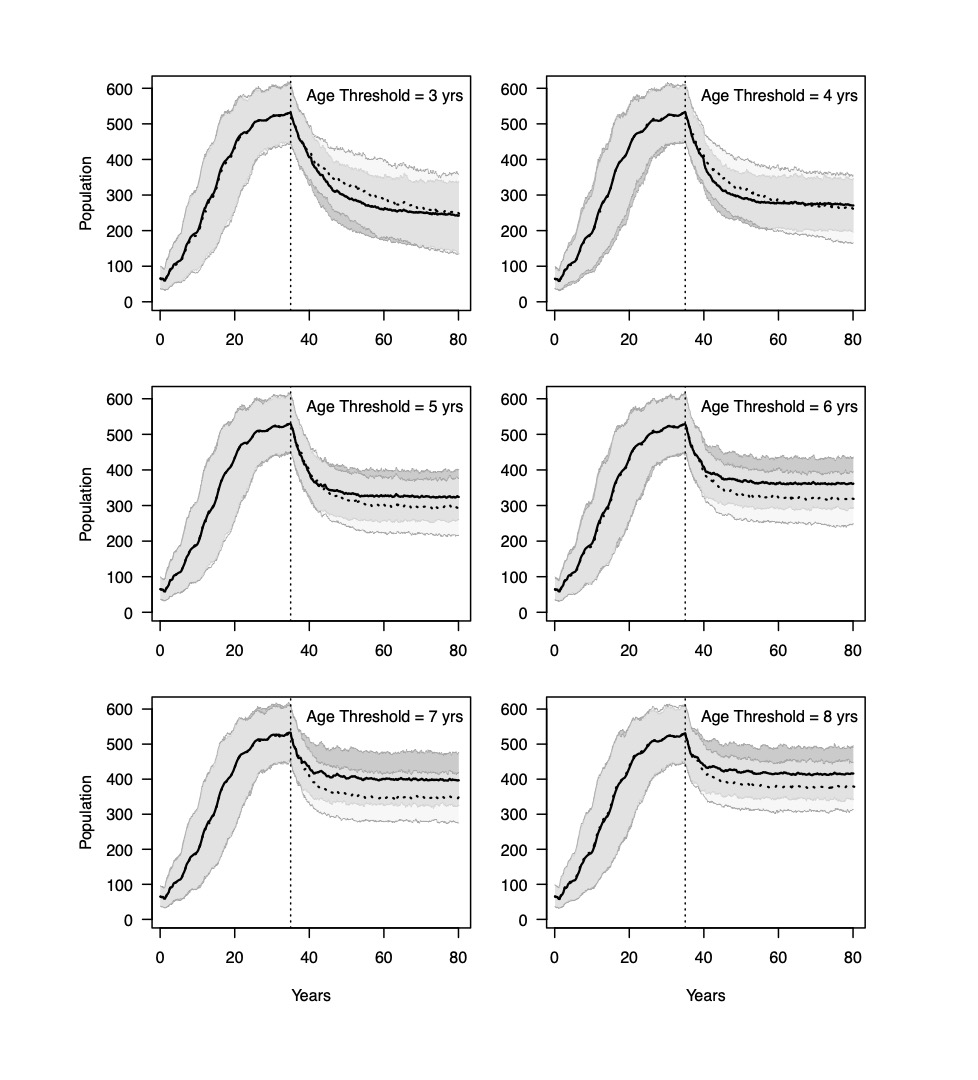


Figure S7. Effect of unreliable age assessment of hunted lions by hunters on lion population persistence for minimum age thresholds of ≥3 to ≥ 8 years. Simulations used an annual hunting quota of 18 males (~1.2 lions/ 1000km^2^) and a monthly conflict mortality rate of 0.4%. 25 of 40 prides in the population affected by edge effects (hunting and conflict). Curves with solid lines denote scenarios where ‘real’ ages of hunted lions were derived from the model and dotted lines incorporate age specific errors in age estimation by hunters. The black lines represent the mean and the light grey areas the 95% confidence interval of simulations. The vertical dotted line marks the point at which test scenarios were implemented after the population had reached its asymptotic state. See Figure S6 for expanded scenarios. After 45 years, populations in estimated age scenarios were the same or smaller than actual age scenarios (% difference between actual and estimated: ≥3 =0, ≥4=0, ≥5= -7.6, ≥6 = -15.7, ≥7 =13.7, ≥8 = 12.9).

We note that with the hunting age threshold set to 3 and 4 years, ageing errors may benefit the lion population over the short term in scenarios without conflict as shown in figures S6a and S6b, though variation is much higher. This likely due to the fact that a greater proportion of individuals are underestimated in age.

**Parameter Elasticity Results**


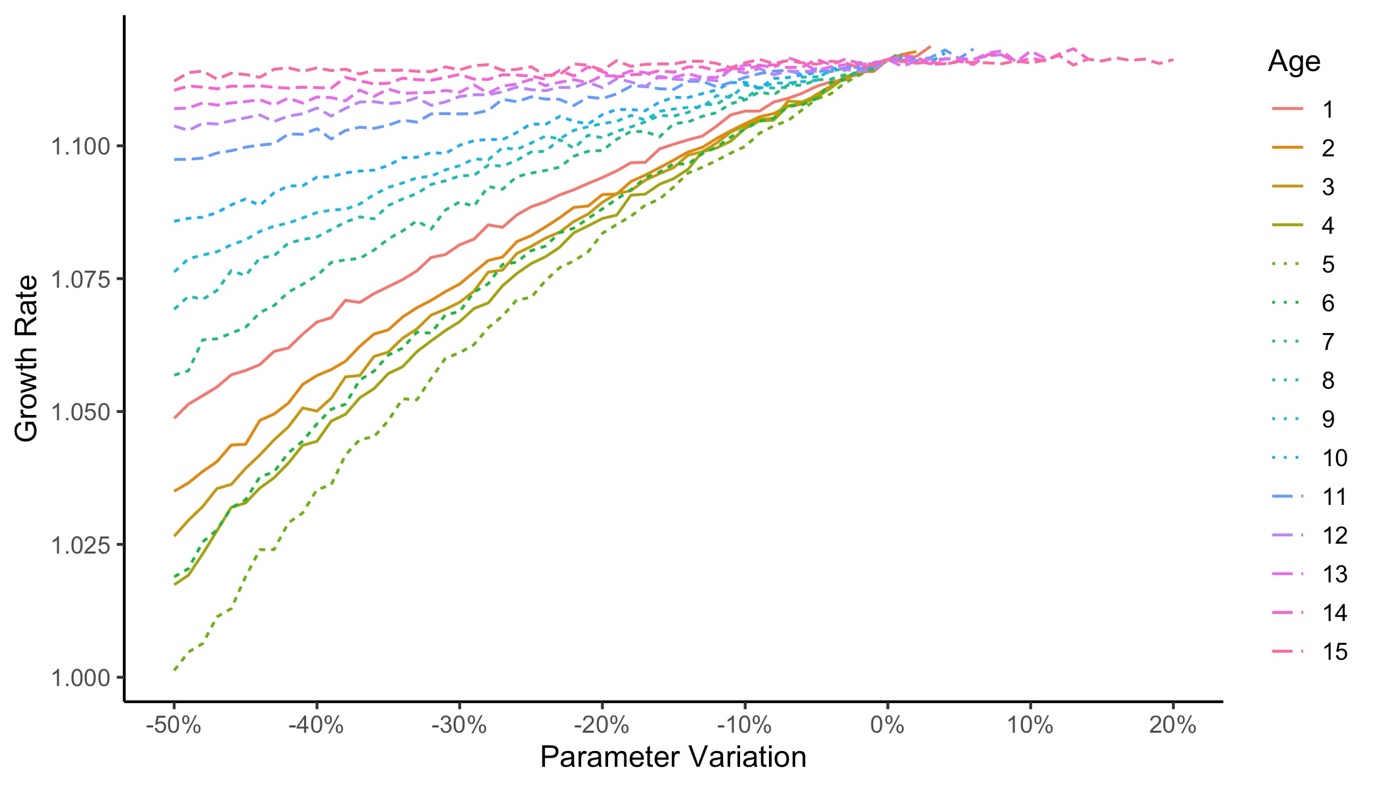


Figure S8. Elasticity of lion population growth rate against changes in age-specific baseline survival probabilities. Different line types represent young (solid line), mature (dotted line) and old (dashed) age individuals. Population growth rate is calculated from year 10 to year 20 of a simulation with 1000 iterations. Population growth rate is more sensitive to declines in survival of individuals that are 4-6 years of age. This age category represents the most reproductively active stage and therefore the impact of lower survival rates is more pronounced. A decrease in survival of individuals > 10 years of age has a relatively small effect on growth rate as there are fewer individuals in this age class within the population which are also less reproductively active. Increases in survival are capped at 1.


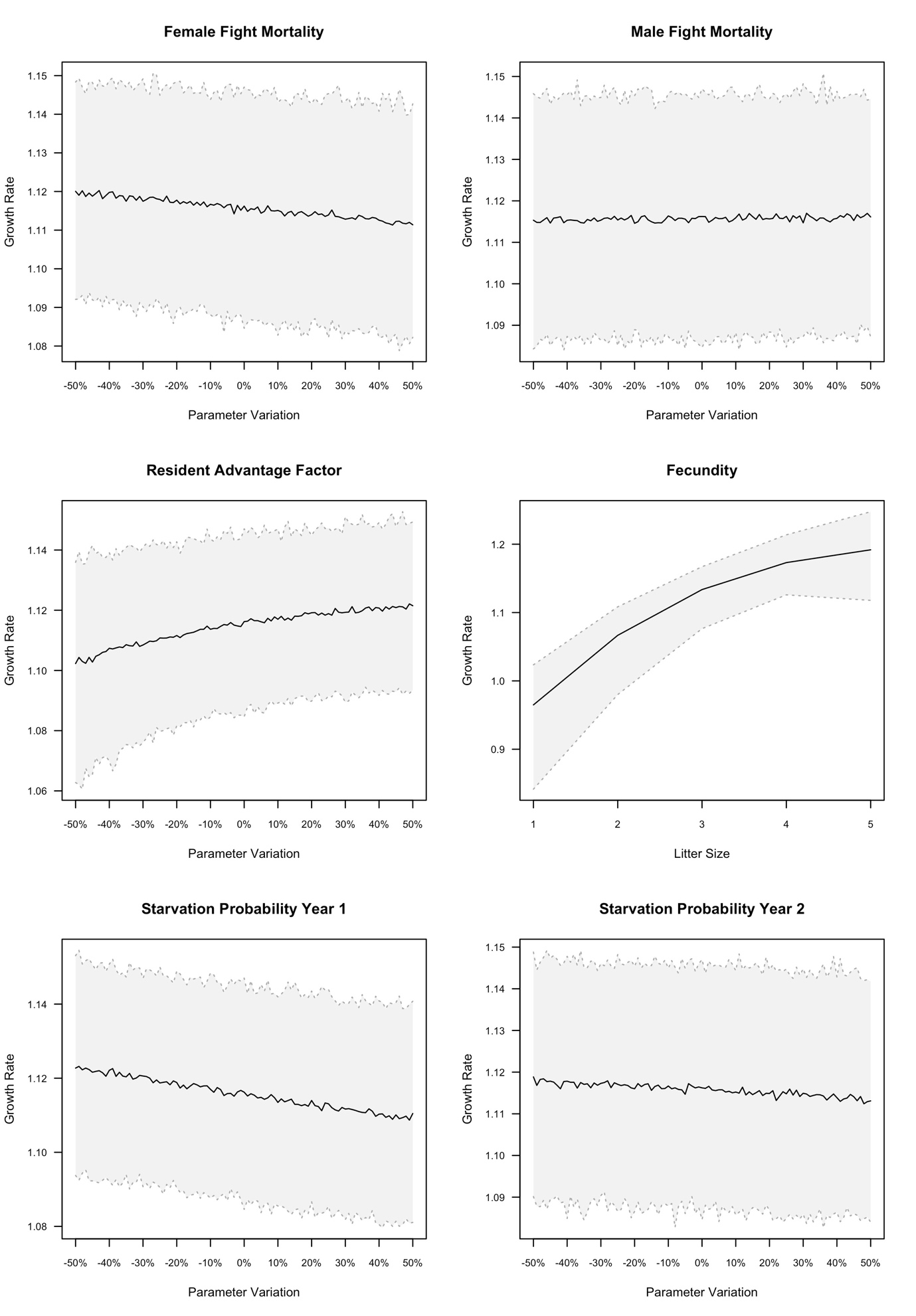


Figure S9. Elasticity of lion population growth rate as a function of changes in fight, fecundity, and starvation parameters. The black lines represent the median and the light grey areas the 95% confidence interval of simulations. Population growth rate is calculated from year 10 to year 20 of a simulation with 1000 iterations. Population growth rate moderately decreases with increases in fight mortalities and starvation mortalities. Population growth rate moderately increases with increasing resident advantage factor. Population growth rate increases with litter size (for this latter parameter, we ran a sensitivity analysis with changes of absolute values – and not relative – as the model uses a multinomial distribution for litter size).

**References**

1 Chapron, G. *et al.* Estimating wolf (Canis lupus) population size from number of packs and an individual based model. *Ecological Modelling* **339**, 33-44, doi:<https://doi.org/10.1016/j.ecolmodel.2016.08.012> (2016).

2 Kernighan, B. W. & Ritchie, D. M. *The C Programming Language*. (Prentice-Hall, 1978).

3 Wilmet, S. The GLib/GTK+ Development Platform. (2014).

4 Grimm, V. *et al.* A standard protocol for describing individual-based and agent-based models. *Ecological Modelling* **198**, 115-126, doi:<https://doi.org/10.1016/j.ecolmodel.2006.04.023> (2006).

5 Morandin, C. *et al.* Gene flow and immigration:genetic diversity and population structure of lions (Panthera leo) in Hwange National Park. *Conservation Genetics* **15**, 697-706, doi:DOI 10.1007/s10592-014-0571-6 (2014).

6 Schaller, G. B. *The Serengeti lion: a study of predator-prey relations*. (Chicago University Press, 1972).

7 Hanby, J. P. & Bygott, J. D. Emigration of sub adult lions. *Animal Behaviour* **35**, 161-169 (1987).

8 Grinnell, J., Packer, C. & Pusey, A. E. Cooperation in male lions: kinship, reciprocity or mutualism? *Animal Behaviour* **49**, 95-105 (1995).

9 West, P. M. *et al.* Wounding, mortality and mane morphology in African lions, *Panthera leo*. *Animal Behaviour* **71**, 609-619 (2006).

10 Whitman, K., Starfield, A. M., Quadling, H. S. & Packer, C. Sustainable trophy hunting of African lions. *Nature* **428**, 175-178 (2004).

11 Whitman, K. L., Starfield, A. M., Quadling, H. & Packer, C. Modeling the effects of trophy selection and environmental disturbance on a simulated population of African lions. *Conservation Biology* **21**, 591-601 (2007).

12 Pusey, A. E. & Packer, C. The evolution of sex-biased dispersal in lions. *Behaviour* **101**, 275-310 (1987).

13 VanderWaal, K. L., Mosser, A. & Packer, C. Optimal group size, dispersal decisions and postdispersal relationships in female African lions. *Animal Behaviour* **77**, 949-954, doi:<https://doi.org/10.1016/j.anbehav.2008.12.028> (2009).

14 Lehmann, M. B., Funston, P. J., Owen, C. R. & Slotow, R. Reproductive biology of a pride of lions on Karongwe Game Reserve, South Africa. *African Zoology* **43**, 230-236, doi:10.1080/15627020.2008.11657239 (2008).

15 Starfield, A. M., Furniss, P. R. & Smuts, G. L. in *Dynamics of Large Mammal populations* (eds C. W. Fowler & T.D. Smith) (Wiley, 1981).

16 Barthold, J., Loveridge, A. J., Macdonald, D. W., Packer, C. & Colchero, F. Bayesian estimates of male and female African lion mortality for future use in population management. *Journal of Applied Ecology* **53**, 295-304 (2016).

17 Barthold, J., Packer, C., Loveridge, A. J., Macdonald, D. W. & Colchero, F. Dead or gone? Bayesian inference on mortality for the dispersing sex. *Ecology and Evolution* **6**, 4910-4923 (2016).

18 Loveridge, A. J. *et al.* Conservation of large predator populations: demographic and spatial responses of African lions to the intensity of trophy hunting. *Biological Conservation* **204**, 247-254, doi:<http://dx.doi.org/10.1016/j.biocon.2016.10.024> (2016).

19 Loveridge, A. J., Valeix, M., Elliot, N. B. & Macdonald, D. W. The landscape of anthropogenic mortality: how African lions respond to spatial variation in risk. *Journal of Applied Ecology* **54**, 815-825, doi:10.1111/1365-2664.12794 (2017).

20 Elliot, N., Cushman, S. A., Macdonald, D. W. & Loveridge, A. J. The devil is in the dispersers. Predictions of landscape connectivity change with demography. *Journal of Applied Ecology* **51**, 1169-1178, doi:doi: 10.1111/1365-2664.12282 (2014).

21 Elliot, N., Valeix, M., Macdonald, D. W. & Loveridge, A. J. Social relationships affect dispersal timing revealing a delayed infanticide in African lions. *Oikos* **123**, 1049-1056, doi:doi: 10.1111/oik.01266 (2014).

22 Loveridge, A. J., Searle, A. W., Murindagomo, F. & Macdonald, D. W. The impact of sport hunting on the population dynamics of an African lion population in a protected area. *Biological Conservation* **134**, 548-558 (2007).

23 Loveridge, A. J., Hemson, G., Davidson, Z. & Macdonald, D. W. in *Biology and Conservation of Wild Felids* (eds D.W. Macdonald & A.J. Loveridge) Ch. 11, 283- 304 (Oxford University Press, 2010).

24 Mbizah, M. *et al.* Effect of ecological factors on fine scale patterns of social structure in African lions. *Journal of Animal Ecology* (2020).

25 Cushman, S. A. *et al.* Prioritizing core areas, corridors and conflict hotspots for lion conservation in southern Africa. *PLOS ONE* **13**, e0196213, doi:10.1371/journal.pone.0196213 (2018).

26 Loveridge, A. J. *et al.* Evaluating the spatial intensity and demographic impacts of wire-snare bush-meat poaching on large carnivores. *Biological Conservation* (2020).

27 Scrucca, L., Santucci, A. & Aversa, F. Competing risk analysis using R: an easy guide for clinicians. *Bone Marrow Transplantation* **40**, 381-387 (2007).

28 Miller, J. R. B. *et al.* Aging traits and sustainable trophy hunting of African lions. *Biological Conservation* **201**, 160-168 (2016).

**Appendix**

***Fight examples***

**Example 1:**

3 x 6-year-old dispersers challenge 2 x 6-year-old resident males:

Chance of RC winning = $\left( \frac{150+150}{100+100+100} \right)^{{(2 - 3)}^{2}+2}$ = 1:1 = 0.5 = 50%

**Example 2:**

1 x 4-year-old & 1 x 5-year-old dispersers challenge 2 x 6-year-old resident males:

Chance of RC winning = $\left( \frac{150+150}{70.7+90.4} \right)^{{(2 - 2)}^{2}+2}$ = 3.47:1 = 0.78 = 78%

**Example 3:**

1 x 3-year-old & 1 x 12-year-old dispersers challenge 2 x 6-year-old resident males:

Chance of RC winning = $\left( \frac{150+150}{52+40.9} \right)^{{(2 - 2)}^{2}+2}$ = 10.4:1 = 0.91 = 91%

**Example 4:**

1 x 7-year-old & 1 x 8-year-old dispersers challenge 1 x 4-year-old, 2 x 5-year-old resident males:

Chance of RC winning = $\left( \frac{106.1+135.6+135.6}{97+92} \right)^{{(3 - 2)}^{2}+2}$ = 8.0:1 = 0.88 = 88%

**Example 5:**

3 x 6-year-old dispersers challenge 1 x 4-year-old, 1 x 5-year-old resident males:

Chance of RC winning = $\left( \frac{106.1+135.6}{100+100+100} \right)^{{(3 - 2)}^{2}+2}$ = 0.52:1 = 0.34 = 34%

**Example 6:**

3 x 6-year-old dispersers challenge 2x 13-year-old resident males:

Chance of RC winning = $\left( \frac{55.5+55.5}{100+100+100} \right)^{{(3 - 2)}^{2}+2}$ = 0.051:1 = 0.05 = 5%
